# Wheat production in Bangladesh: its future in the light of global warming

**DOI:** 10.1093/aobpla/pls042

**Published:** 2012-11-11

**Authors:** Akbar Hossain, Jaime A. Teixeira da Silva

**Affiliations:** 1 Wheat Research Center, Bangladesh Agricultural Research Institute, Dinajpur 5200, Bangladesh; 2 Faculty of Agriculture and Graduate School of Agriculture, Kagawa University, Ikenobe, Miki-cho 761-0795, Japan

## Abstract

**Background and aims:**

The most fundamental activity of the people of Bangladesh is agriculture. Modelling projections for Bangladesh indicate that warmer temperatures linked to climate change will severely reduce the growth of various winter crops (wheat, boro rice, potato and winter vegetables) in the north and central parts. In summer, crops in south-eastern parts of the country are at risk from increased flooding as sea levels increase.

**Key facts:**

Wheat is one of the most important winter crops and is temperature sensitive and the second most important grain crop after rice. In this review, we provide an up-to-date and detailed account of wheat research of Bangladesh and the impact that global warming may have on agriculture, especially wheat production. Although flooding is not of major importance or consequence to the wheat crop at present, some perspectives are provided on this stress since wheat is flood sensitive and the incidence of flooding is likely to increase.

**Projections:**

This information and projections will allow wheat breeders to devise new breeding programmes to attempt to mitigate future global warming. We discuss what this implies for food security in the broader context of South Asia.

## Abstract (precis)

Global warming has already seen a radical change in temperature regimes in Bangladesh. This review provides the first up-to-date perspective and detailed analysis of wheat research in Bangladesh and the impact that global warming will have on its agriculture, especially wheat farming.

## Introduction

### History of wheat production in Bangladesh

**Fig. 1:**
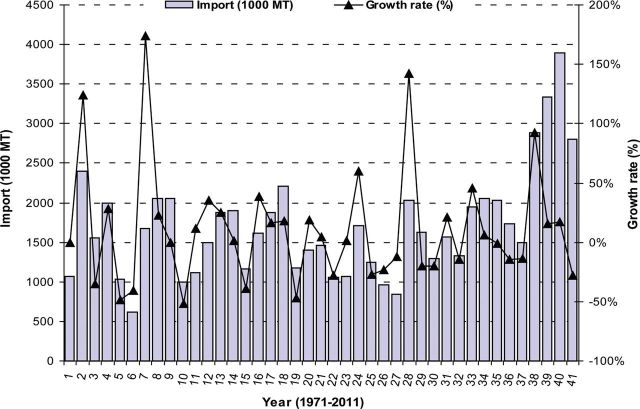
**Yearly import (1000 MT) and growth rate (%) of wheat from 1971 to 2011 in Bangladesh.** Growth rate (%) was calculated relative to the previous year's value (data source: Index Mundi 2012*b*).

**Fig. 2:**
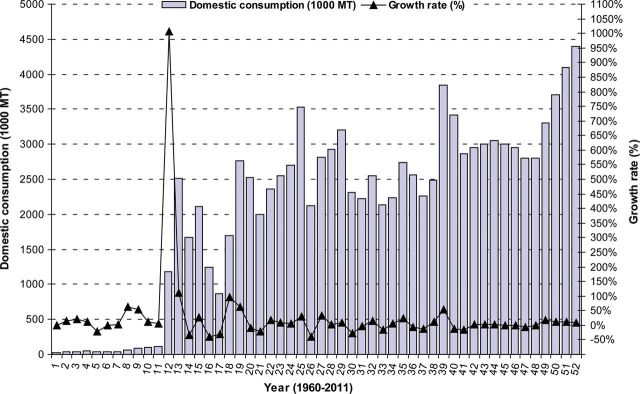
**Domestic consumption (1000 MT) and growth rate (%) of wheat from 1960 to 2011 in Bangladesh.** Growth rate (%) was calculated relative to the previous year's value (data source: Index Mundi 2012*b*).

Immediately after independence in 1971, a series of disastrous harvests (attributable largely to unfavourable weather) led to widespread food shortages in Bangladesh. This forced the government to appeal to the international community for emergency relief assistance. Massive imports of cereals, edible oils and dairy products became a regular feature of the economy, and Bangladesh developed a reputation as one of the world's most impoverished nations (IFPRI 1997). From March to December 1974, Bangladesh faced an acute food shortage as the price of rice increased sharply in the world market (OECD-FAO 2009) and production decreased (Alamgir 1980). World rice prices increased sharply from 1971 to 1975, resulting in food shortages in Bangladesh (OECD 2008). Rice production declined (Index Mundi 2012*a*) because of disruptions to virtually all agricultural activities during the War of Liberation in 1971 and various natural disasters, such as floods, droughts, cyclones and rapid population growth (Sobhan 1979; Alamgir 1980; Sen 1982; Hugo 2006). At that time, it was realized that rice alone could not meet the food requirements of the country (Banglapedia 2006). Wheat was therefore chosen as an alternative winter food crop. Two Mexican varieties (‘Sonora 64’ and ‘Penjamo 62’) were tested first in the northern part of Bangladesh in 1965 (BARI 2010). Their spectacular performance encouraged scientists to introduce wheat more generally to this part of the country. By the time of independence (1971), Bangladesh had become highly dependent on wheat imports while dietary preferences were changing such that wheat was becoming a highly desirable food supplement to rice. In the first half of the 1980s, domestic wheat production rose to more than 1 million tons year^−1^, but was still only 7–9 % of total food grain production (BARI 2010). About half of wheat was grown on irrigated land and the proportion of land devoted to wheat remained essentially unchanged between 1980 and 1986, at a little less than 6 % of the total planted area (Index Mundi 2012*b*). Wheat also accounted for the greatest bulk of imported food grains, exceeding 1 million tons annually and rising above 1.8 million tons in 1984, 1985 and 1987 (Index Mundi 2012*b*). The great bulk of wheat importation is financed under aid programmes of the USA, the European Union and the World Food Programme (Index Mundi 2012*b*). A 3-year (2008–09 to 2010–11) examination by O'Brien (2011) indicated that Bangladesh imported 3.1 million metric tons of wheat each year to ensure local demand. By utilizing Index Mundi (2012*b*) data, we show wheat import in Fig. 1 and domestic consumption specific to Bangladesh in Fig. 2.

### Past and present status of production programme in Bangladesh

**Fig. 3:**
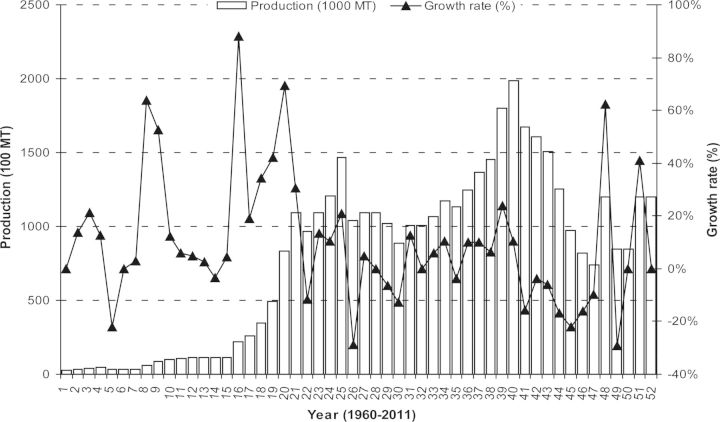
**Yearly production (1000 MT) and growth rate (%) of wheat from 1960 to 2011 in Bangladesh.** Growth rate (%) was calculated relative to the previous year's value (data source: Index Mundi 2012*b*).

**Fig. 4:**
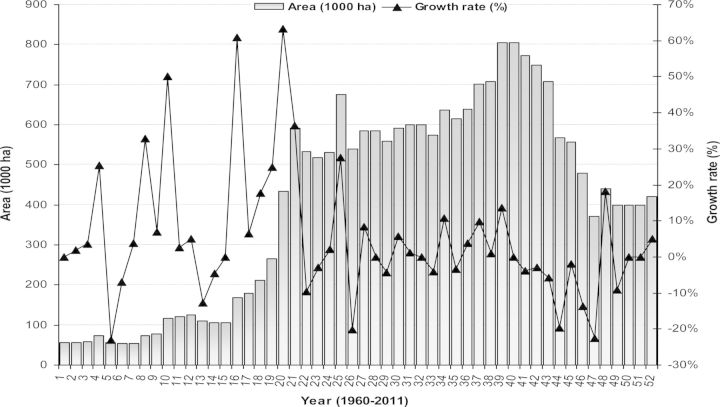
**Area cultivated (1000 ha) and growth rate (%) of wheat from 1960 to 2011 in Bangladesh.** Growth rate (%) was calculated relative to the previous year's value (data source: Index Mundi 2012*b*).

Initially, 4000 tons of ‘Sonalika’ and ‘Kalyansona’ seeds were imported from India in 1975 and distributed to farmers (BARI 2010). Prior to 1975–76, wheat was grown sporadically and was almost an unknown crop in Bangladesh (Banglapedia 2006). Between 1970–71 and 1980–81, the cropped area under wheat jumped from 0.126 million ha to 0.591 million ha and production rose 10-fold from 0.11 million tons to 1.07 million tons, a 24.93 % annual mean growth rate (BARI 2010). Among the cereals, wheat is second to rice in economic and consumption importance (Fig. 2). It occupies ∼4 % of the total cropped area and 11 % of the area cropped in rabi (winter crops starting from November to February), and contributes 7 % to the total output of food cereals (Anonymous 2008). By collecting 52 years of data from Index Mundi (2012*b*), we show the trend of area, production and growth rate of wheat in Bangladesh from 1960 to 2011 (Figs 3 and 4).

**Fig. 5:**
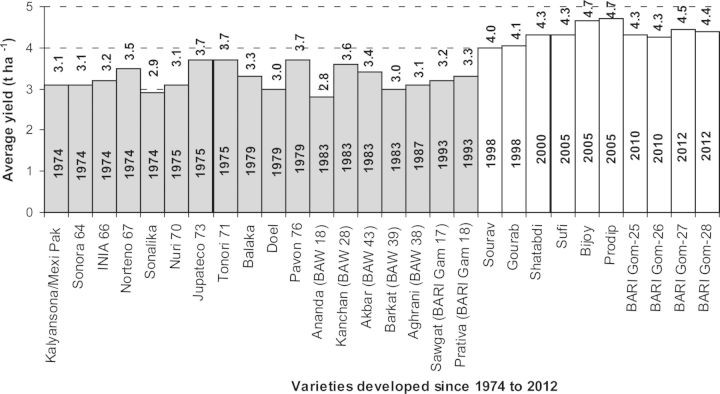
**Year of release and average yield (tons ha^−1^) of Bangladesh wheat varieties developed since 1974 up until 2012**. Figures in the centre of the bars indicate the year of release, while those above the bars indicate average yield (tons ha^−1^). Bars in white indicate existing Bangladesh wheat varieties (i.e. bred in BARI) (data source: BARI 2012*a*).

**Fig. 6:**
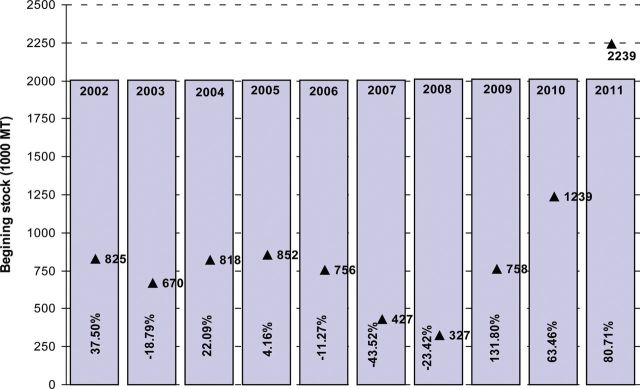
**Beginning stock (▴ 1000 MT) for future security and growth rate (%) of wheat over the last 10 years (2002–2011) in Bangladesh.** Bars indicate years from 2002 to 2011. The percentage values (±) in the bars indicate the growth rate relative to the previous year (data source: Index Mundi 2012*b*).

From 52 years' data it was observed that, in the last 5 years, increased domestic consumption of wheat (Fig. 2) can be linked to increases in population (Anonymous 2011) and changes in eating habits (WRC 2009). Even though existing wheat varieties in Bangladesh are high yielding (Table 1; Fig. 5), area (Fig. 4) and production (Fig. 3) did not increase sufficiently to match the growth in the human population. Moreover, many wheat crops were replaced by different rabi crops such as potato, boro rice, maize and different short-duration vegetables (WRC 2009). In this situation, to meet the demands of an increasing population and to secure future food security (initial stock indicated in Fig. 6), the government of Bangladesh imported more wheat between 2008 and 2011 than it did in previous years (Fig. 1).

**Table 1:** Characteristics of existing wheat varieties of Bangladesh developed since 1998 (BARI 2012*a*).

Variety	Stress tolerance capacity	Life duration (days)	Yield (kg ha^−1^)	Suitable area for cultivation	Year of release	Sowing time	Harvest time	Major diseases and pests
Sourav	Moderately heat tolerant	102–110	3500–4500	All over the country except saline areas	1998	Nov. 15–30	Mar.–Apr.	Tolerant to *Bipolaris* leaf blight and resistant to leaf rust
Gourab	Heat sensitive	102–108	3500–4600	All over the country except saline areas	1998	Nov. 15–30	Mar.–Apr.	Tolerant to *Bipolaris* leaf blight and resistant to leaf rust
Shatabdi	Good level of tolerance to terminal heat	105–110	3600–5000	All over the country except saline areas	2000	Nov. 15–30	Mar.–Apr.	Highly tolerant to *Bipolaris* leaf blight and resistant to leaf rust
Sufi	Tolerant to late heat stress	105–110	3600–5000	All over the country except saline areas	2005	Nov. 15–30	Mar.–Apr.	Highly tolerant to *Bipolaris* leaf blight and resistant to leaf rust
Bijoy	Moderately heat tolerant	103–112	4300–5000	All over the country except saline areas	2005	Nov. 15–30	Mar.–Apr.	Highly tolerant to *Bipolaris* leaf blight and resistant to leaf rust
Prodip	High yielding, but heat sensitive	102–110	4300–5100	All over the country except saline areas	2005	Nov. 15–30	Mar.–Apr.	Highly tolerant to *Bipolaris* leaf blight and resistant to leaf rust
BARI Gom 25	Moderate level of tolerance to heat stress	102–110	3600–5000	Suitable for southern region (8–10 dS m^−1^ salinity level)	2010	Nov. 15–30	Mar.–Apr.	Highly tolerant to *Bipolaris* leaf blight and resistant to leaf rust
BARI Gom 26	Tolerant to terminal heat stress in late seeding	104–110	3500–5000	Possible to grow throughout the country except in areas with salinity level >6 dS m^−1^	2010	Nov. 15–30	Mar.–Apr.	Tolerant to *bipolaris* leaf blight and resistant to leaf rust (stem rust) race Ug99
BARI Gom 27	Moderate level of tolerance to heat stress	105–110	3800–5400	All over the country except saline areas	2012	Nov. 15	Mar.–Apr.	It is resistant to leaf rust and tolerant to *Bipolaris* leaf blight and possesses good level of APR to the Ug99 race of stem rust and its variants
BARI Gom 28	Tolerant to terminal heat stress in late seeding	100–105	4000–5500	All over the country except saline areas	2012	Nov. 15	Mar.–Apr.	It is resistant to leaf rust and tolerant to *Bipolaris* leaf blight

### Introduction of high-yielding varieties

In the initial stages of wheat growing in Bangladesh, several Mexican varieties, especially ‘Sonora 64’ and ‘Kalyansona’, were successfully introduced in collaboration with the International Maize and Wheat Improvement Center (CIMMYT). However, the release of ‘Sonalika’ in 1972 created a true breakthrough in wheat production. This fast maturing and high-yielding variety (yield = 2 tons ha^−1^) became very popular among wheat growers and adapted well to different production environments, and was adopted in 80 % of the wheat area by the early 1980s (WRC 2009). In 1983, the Wheat Research Centre (WRC), Bangladesh Agricultural Research Institute (BARI), released four more high-yielding (yield = 2–3 tons ha^−1^) varieties (‘Ananda’, ‘Kanchan’, ‘Barkat’ and ‘Akbar’). Among these, ‘Kanchan’ proved particularly adaptable and gradually replaced ‘Sonalika’ to become the predominant variety in Bangladesh by the early 1990s. Two other high-yielding varieties, ‘Aghrani’ and ‘Protiva’, were recommended by the Bangladesh National Seed Board in 1987 and 1993, respectively. These varieties were more responsive to a wider range of weather conditions as well as crop management practices such as fertilizers, irrigation and intercultural operations. Therefore, by the mid-1990s, adoption of high-yielding varieties was almost 100 %, thereby increasing wheat productivity substantially. The year of release and average yield of the new wheat varieties developed in Bangladesh from 1974 to 2012 are presented in Fig. 5.

However, breeding efforts to develop high-yielding varieties still continued. Several more high-yielding varieties were developed. These included ‘BARI Gom 19’ (‘Sourav’) and ‘BARI Gom 20’ (‘Gourab’) released in 1998; ‘BARI Gom 21’ (‘Shatabdi’) in 2000; ‘BARI Gom 22’ (‘Sufi’), ‘BARI Gom 23’ (‘Bijoy’) and ‘BARI Gom 24’ (‘Prodip’) in 2005 (Pandit *et al*. 2011); and ‘BARI Gom 25’ and ‘BARI Gom 26’ released in 2010 (BARI 2012*b*). In 2012, two more varieties, ‘BARI Gom 27’ and ‘BARI Gom 28’, were released. Some general characteristics of the existing elite wheat varieties of Bangladesh are presented in Table 1. Thanks largely to the new varieties, total production increased between 1970 and 2002, but thereafter production decreased as the production area also fell (based on 52 years of data from Index Mundi (2012*b*); Figs 3 and 5). The decrease in area was due to competition from other rabi-grown crops such as boro rice, potato and maize (WRC 2009), even though the yield of existing wheat varieties increased through intensive research (Table 1; Fig. 5).

### International collaboration

The CIMMYT has worked closely with the Bangladesh wheat programmes and has played a vital role in popularizing wheat cultivation in Bangladesh from the start. The CIMMYT provided an enormous elite wheat germplasm from which promising types could be selected to suit the Bangladesh environment. Many wheat scientists attended in-service training at CIMMYT on issues such as wheat improvement, production agronomy, wheat quality and station management (Pandit *et al*. 2011). Some Bangladesh Agricultural Development Corporation (BADC) personnel were also trained on seed production by CIMMYT. Such training programmes were initiated in 1969 and still continue. The CIMMYT wheat scientists visit research stations and farmers' fields. This collaboration was a key factor in quickly turning Bangladesh into a wheat-growing country. In addition to collaboration with CIMMYT, the Canadian International Development Agency (CIDA), Australian Government Overseas Aid Program (Aus-AID), United States Agency for International Development (USAID) and the Ford Foundation provided grants for facility improvement and manpower development of the WRC, BARI, in Bangladesh (Pandit *et al*. 2011).

## Global warming and its impact on world wheat production

### Climate change and its impact on global wheat production

Recurrent food crises combined with the recent global financial problems, volatile energy prices, natural resource depletion and climate change have combined to undercut and threaten the livelihoods of millions of poor people worldwide. Wheat accounts for a fifth of humanity's food and is second only to rice as a source of calories in the diets of consumers in developing countries and is first as a source of protein (Braun *et al*. 2010). Wheat is an especially critical foodstuff for ∼1.2 billion people classified as ‘wheat-dependent’; 2.5 billion are classified as ‘wheat-consuming’ and live on <US$2 day^−1^. There are also ∼30 million poor wheat producers and their families for whom wheat is the staple crop (FAOSTAT 2012; Table 2). Demand for wheat in the developing world is projected to increase 60 % by 2050 (Rosegrant and Agcaoili 2010). The International Food Policy Research Institute projections indicate that world demand for wheat will rise from 552 million tons in 1993 to 775 million tons by 2020 (Rosegrant *et al*. 1997). At the same time, climate change-induced temperature increases are likely to reduce wheat production in developing countries (where around 66 % of all wheat is produced) by 20–30 % (Esterling *et al*. 2007; Lobell *et al*. 2008; Rosegrant and Agcaoili 2010). The Intergovernmental Panel on Climate Change (IPCC) (2007) noted that global climate change will have a major impact on crop production. CIMMYT and ICARDA (2011) estimated that 20–30 % wheat yield losses will occur by 2050 in developing countries as a result of a predicted temperature increase of 2–3 °C. On a global scale, these yield losses will not be fully compensated by yield gains in high-latitude regions (Canada, Russia, Kazakhstan and Northern USA), estimated at 10–15 % (OECD-FAO 2009), since major wheat producers such as France have already reported yield reductions due to increasing temperatures (Charmet 2009).

**Table 2:** Global and regional wheat production and consumption statistics in wheat-producing countries (FAOSTAT 2012).

Subregion	Area (million ha)	Production (million tons)	Yield (kg ha^−1^)	Population (million)	Population earning <1$/day (%)	Population earning <2$/day (%)	Million people earning <2$/day (%)	Kcal/capita/day
Eastern Asia	23.9	110.4	4628	1406	16	45	634	597
Southern Asia	37.2	98.7	2656	1542	32	79	1212	481
Central Asia	16.1	26.4	1633	78	3	31	24	1279
Middle East/North Africa	26.8	61.6	2296	514	6	23	120	1154
Eastern Africa	1.7	2.9	1735	229	26	70	160	192
Southern Africa	0.9	2.3	2934	156	11	21	33	258
Western Africa	0.1	0.1	1478	184	69	90	165	135
South America	8.4	20.7	2464	385	9	24	92	430
Central America	0.7	3.6	5065	131	11	29	37	264
North America	30.2	82	2728	341	0	0	0	603
Eastern Europe	38.7	100	2587	295	0	0	0	963
North and western Europe	13.2	90.1	6820	287	0	0	0	701
Southern Europe	5.8	19.6	3364	154	0	1	1	836
Australia/New Zealand	12.6	15	1222	26	0	0	0	547
Total	216.2	634	2933	5727	18	43	2478	597

### Expected changes in wheat-growing areas in South Asia

The CIMMYT has recently examined the potential impact of climate change on wheat production. For the purposes of crop technology development, CIMMYT classifies world production in terms of ‘mega-environments’ (Consultative Group on International Agricultural Research (CGIAR) 2009). These are broad, often non-contiguous or even transcontinental areas showing similar crop production conditions. Focusing on South Asia's Indo-Gangetic Plain, which produces ∼90 million tons of wheat grain year^−1^ (or nearly 15 % of total production worldwide), CIMMYT researchers examined how climate change might affect the current classification of two wheat mega-environments (CGIAR 2009). While both are irrigated, one is favourable for wheat production, while the other is not because of heat stress early and late in the growing season. Under the climatic conditions expected to prevail by 2050, researchers project that the favourable wheat mega-environment will shrink by just over half, mainly because of increased heat stress (CGIAR 2009). This will most probably lead to a major reduction in wheat harvests, threatening the food security of ∼200 million people (CGIAR 2009).

Regarding agriculture and food security overall, crop yields of wheat, maize and rice are predicted to decrease in South Asia by up to 30 % by the end of this century (compared with an increase of up to 20 % in East and South East Asia) (Anonymous 2012). In cereal production alone, the most conservative climate change projections suggest a minimum decline across South Asia of between 4 and 10 %. In Bangladesh, rice production could fall by 8 % and wheat production by 32 % as early as 2050 (Anonymous 2012). Substantial losses in rain-fed wheat are also anticipated. Studies in India suggest that a 0.5 °C rise in winter temperature would reduce wheat yield by 0.45 tons ha^−1^. Similarly, a rise in temperature beyond 2.5 °C would reduce non-irrigated wheat and rice farm revenue by 9–25 % (Anonymous 2012).

### Future strategies to combat the impact of global warming on wheat production

In response to the predicted problems for wheat production summarized above, researchers in numerous countries are trying to develop heat-tolerant wheat varieties or improve management practices to mitigate the effects of future global warming. Representative research findings related to high temperature, drought stress and their effect on different wheat cultivars in different countries around the world are presented in Table 4.

## Global warming and its impact on wheat production in Bangladesh

### Change of temperature in Bangladesh due to global warming

The Geophysical Fluid Dynamics Laboratory transient model (Manabe *et al*. 1991) projected that, in Bangladesh, temperatures would rise 1.3 °C by 2030 and 2.6 °C by 2070, compared with mid-20th-century levels. These values are slightly above those given in Table 3 and may reflect lower climate sensitivity in more recent climate models. The core findings, however, are consistent with the analysis presented above. The report estimated that winter warming would be greater than summer warming. The study also estimated little change in winter precipitation and an increase in precipitation during the monsoon season (Ahmed and Alam 1999). On the other hand, the annual mean temperature of Bangladesh is 25.75 °C and is expected to rise by 0.21 °C by 2050 (Karmakar and Shrestha 2000). Karttenberg *et al*. (1995) stated that crops may be exposed to more thermal stress in the near future since global warming is expected to increase temperatures by 2 °C by the middle of the 21st century. The Organization for Economic Co-operation and Development (OECD) estimated a rise in temperature of 1.4 °C by 2050 and 2.4 °C by 2100 in Bangladesh (OECD 2003; Table 3). The current assessment for Bangladesh by the IPCC (2007) predicts warming of 1.5–2.0 °C by 2050, with 10–15 % increased rainfall by 2030 and a 12 % increase in evaporation by 2030. Using data from 34 meteorological climate sites in Bangladesh, A. S. Islam (2009) and C. M. A. Islam (2009) estimated that maximum and minimum February temperatures had increased by 0.62 and 1.54 °C, respectively, over the past 100 years for all of Bangladesh. Poulton and Rawson (2011) reported that temperature in Bangladesh increased over the past two decades at 0.035 °C year^−1^. If this trend continues, by 2050, temperatures will have increased over 1990 levels by 2.13 °C. Yusuf *et al*. (2008) stated that between 1961 and 2007, mean south-west monsoon (June–October) as well as post monsoon (October–November) temperature increased by 0.8 °C. They also noticed that annual mean maximum temperature had risen by 0.6 °C while, more alarmingly, both the annual mean minimum and the winter (December–February) mean minimum temperature increased by 0.3 °C over the same period.

**Table 3:** General circulation model estimates of temperature and precipitation changes (source: OECD 2003).

Year	Temperature change (°C) mean			Precipitation change (%)		
	Annual	DJF	JJA	Annual	DJF	JJA
Baseline average				2278 mm	33.7 mm	1343.7 mm
2030	1.0 (0.11)	1.1 (0.18)	0.8 (0.16)	+3.8 (2.30)	−1.2 (12.56)	+4.7 (3.7)
2050	1.4 (0.16)	1.6 (0.26)	1.1 (0.23)	+5.6 (3.33)	−1.7 (18.15)	+6.8 (4.58)
2100	2.4 (0.28)	2.7 (0.46)	1.9 (0.40)	+9.7 (5.8)	−3.0 (31.60)	+11.8 (7.97)

Figures in parenthesis indicate standard deviation.

DJE, December, January and February; JJA, June, July and August.

Bangladesh is a deltaic land of ∼144 000 km^2^ bordered by the Himalayas to the north and the Bay of Bengal to the south. Its South Asian position extends from 20°45′N to 26°40′N and from 88°05′E to 92°40′E. Fifty per cent of the country's land elevation is within 5 m of sea level. About 68 % of the country is vulnerable to flooding while 20–25 % of the area is inundated during normal flooding. It has a complex coastline of ∼710 km and a long continental shelf with a shallow bathymetry. The Bay of Bengal forms a funnel shape towards the Meghna estuary. Because of this, its storm surge is the highest in the world (A. S. Islam 2009; C. M. A. Islam 2009). Water-stressed wheat, in response to flooding, suffers considerable changes in its metabolic profile, particularly proteins (Noorka and Teixeira da Silva 2012). Flooding can therefore alter the nutritional profile of this crop.

Existing vulnerability to flooding, caused by upstream deforestation, will be aggravated by the effects of climate change. Climate change is anticipated to bring increased sedimentation and an increase in extreme rainfall events, both of which will increase flood risk beyond its current high levels, as witnessed by Cyclone Sidr in 2007, the most devastating cyclone to have struck Bangladesh (Haq *et al*. 2012). All coastal areas in Asia are currently facing increasing stress with threats to human and environmental resilience. However, rising sea levels will have a further, major impact on coastal and low-lying communities in South Asia. The most conservative climate change scenarios predict a rise in sea level of 40 cm by the end of this century, which will increase the annual number of people affected by flooding in Asia from 12 million to 94 million, with almost 60 % of these people living in South Asia (including the coastlines of Pakistan, Sri Lanka and Bangladesh). Modelling suggests that 1 million people will be directly affected by a rise in sea level in 2050 in the region of the Bangladesh Ganges–Brahmaputra–Meghna mega-delta. Moreover, Bangladesh's coastal areas will continue to suffer from saline water intrusion, coastal land degradation, storm surges and drainage congestion due to high water flow and sedimentation in the flood plain (Rahman 2011; Practical Action 2012).

### Future climate change and its impact on agriculture of Bangladesh

Major basic food in the Bangladeshi diet comprises rice, wheat, pulses, potato, vegetables and fish. These foods contribute almost 85 % of the total calorie and protein intake (Begum and Luc D'Haese 2010). Rice and wheat alone contribute 71 and 53 % of the total per capita calorie and protein intake, respectively (Anonymous 2008). It was projected that in the years up to 2021, annual demand for food exceeds supply. The shortfall was −0.28 % for rice and −1.76 % for wheat. This implies that demand is greater than supply for both crops (Begum and Luc D'Haese 2010).

Pressure for increased crop production is generated mostly by rapid population growth. This constitutes a most serious challenge to political and social strategists. Agriculture is also a major employer (44 % of the total workforce) and contributes ∼12 % of Bangladesh's GDP (IUCN 2012). For these reasons, the government has given topmost priority to the agriculture sector. This is directly related to the relief of rural poverty since agriculture benefits the livelihood of rural poor people who account for the majority of the population. The agriculture sector is therefore the primary income contributor and employment generator in Bangladesh (Planning Commission 2009). Between 2000 and 2005, the prevalence of poverty in Bangladesh diminished from 49 % to 40 % (56 million). However, by mid-2008, an additional 8.5 % (i.e. 12 million) were feared to have slid below the poverty line due to rises in food prices in the world market. The prevalence of poverty is now ∼48.5 %, as reported by the CPD (Centre for Policy Dialogue 2012). Bangladesh is one of the most climate-vulnerable countries in the world. Located between the Himalayas and the Bay of Bengal, the country is very prone to natural disasters (World Bank 2009). Climate change accelerates the intensity and frequency of these through increasing salinity, storms, drought, irregular rainfall, high temperature, flash floods, etc., which are presumed to be the results of global warming.

Every crop has an optimal temperature range for vegetative and reproductive growth. When the temperature falls below this range or exceeds the upper limit, then that crop production faces constraints. Islam *et al*. (2008) found that a 10 °C increase in maximum temperature at vegetative, reproductive and ripening stages caused a decrease in aman rice production by 2.94, 53.06 and 17.28 tons, respectively. With a change in average temperature of 2–4 °C, the prospect of growing wheat and potato would be severely impaired and production loss may exceed 60 % of the achievable yields (Karim 1993). On the other hand, the response of soil organic matter (SOM) decomposition to increasing temperature is a critical aspect of an ecosystem's responses to global change. The temperature sensitivity of soil carbon decomposition is a key factor determining the response of the terrestrial carbon balance to climatic change, as most recently shown in coupled global carbon climate–vegetation model studies (e.g. Jones *et al*. 2003). Consequently, the temperature sensitivity of soil respiration and SOM decomposition has received much attention (e.g. Luo *et al*. 2001; Reichstein *et al*. 2003; Sandermann *et al*. 2003; Leite and Madari 2011).

An increase in winter temperature can reduce the environmental suitability for wheat, potato and other temperate crops grown in rabi in the north and central parts of the country (A. S. Islam 2009; C. M. A. Islam 2009). Braun *et al*. (2010) reported that while accounting for a fifth of human food consumption, wheat is second only to rice as a source of calories in developing countries such as Bangladesh, and is first as a source of protein. With over 35 % of Bangladeshis suffering from malnourishment (Lal *et al*. 2001), the threat of increased hunger from a reduction in agricultural production would suggest agriculture as one of the major vulnerabilities facing the country.

Climate change is already thought to be increasing the incidence and intensity of droughts: there were only five devastating droughts in the 100 years from 1800 to 1900, yet, since 1981, four major droughts have occurred in the last 25 years, mostly in north-western Bangladesh (Selvaraju *et al*. 2006). The area affected is also expected to grow. For example, the area severely affected by rabi droughts could increase from 4000 to 12 000 km as global warming increases (Huq 2006). Devastating and regular droughts caused by a lack or a late/early arrival of rainfall are common in many parts of Bangladesh. The impact of drought, associated with late or premature monsoon rains or even complete failure of monsoon, spreads over a much larger geographical area than areas affected by other natural hazards. Bangladesh experienced major droughts in 1973, 1978–79, 1981–82, 1989, 1992 and 1994–95. Food grain production lost in the 1978–79 drought was probably 50–100 % more than that lost in the great flood of 1974, directly affecting 42 % of cultivated land. This shows that drought can be as devastating as major floods or cyclones. Rice, jute and other crops were greatly affected, and livestock also suffered from the severe water shortage. More recently, the droughts of 1994–95 in the north-western districts of Bangladesh led to a 3.5-million-ton shortfall of rice and wheat production, while the 1997 drought caused an ∼1-million-ton shortfall of food grain, of which ∼0.6 million tons was transplanted with aman rice valued at around US$500 million (Selvaraju *et al*. 2006).

Bangladesh ranks sixth among the countries in the world most vulnerable to floods. Owing to its topography and position, the country is experiencing flooding in 30–50 % of the area and almost every year. These extreme weather events cause heavy losses to agricultural production, particularly to broad acre crops. Flooding has become severe in the past 4–5 years with severe flooding inundating roughly 60 % of the country. The effect was especially severe on crop agriculture and thus on the livelihood of most Bangladeshi people (Rahman 2011). The reductions in yields of staple crops such as rice and wheat caused by flooding are anticipated to be very great. By 2050, rice yield could drop by 8 % and wheat yields by 32 % (Practical Action 2012). Beyond these values and predictions, there are no further statistics to help manage wheat cropping under flooded conditions. Furthermore, no flooding-tolerant varieties have yet been developed by Bangladeshi scientists, although a start has been made recently. Less attention is given to the effect of flooding on wheat compared with rice since wheat is cultivated mostly in the highlands in the north.

## Strategies to decrease the impact of global warming on wheat production

As a result of intensive research and breeding, the yield potential and yield quality of existing wheat varieties of Bangladesh have improved. Average yields now stand at 4.0–4.7 tons ha^−1^ (Fig. 5). The majority of varieties grown are sensitive to high temperature, and yield safety is in jeopardy because of the forecast climatic changes. Drought and high temperature are key stress factors with high potential for impacting negatively on crop yield. Yield safety can only be improved if future breeding is based on new knowledge concerning plant development and its responses to stress, for example by enabling the development of crop plants with improved thermo-tolerance using various genetic approaches (Wahid *et al*. 2007). A thorough understanding of physiological responses of plants to high temperature, mechanisms of heat tolerance and possible strategies for improving crop thermo-tolerance will be needed for this. In this context, Bangladesh researchers are breeding heat- and drought-tolerant cultivars, and developing new technologies comparable with those in more advanced countries (Table 4), in collaboration with various national and international organizations (i.e. CIMMYT, ICARDA, ICRISAT, CIDA, Aus-AID, USAID). These contributions are summarized in Tables 5 and 6.

**Table 4:** Recent studies related to heat, drought and low-temperature stress in different countries around the world (chronological order).

Country	Tested cultivars	Main research findings	Reference
Denmark	Tested 2255 Mexican wheat landraces	Landraces were evaluated on the basis of canopy temperature depression (LCC), and 1000-kernel weight. Three landrace cultivars with superior and consistent LCC values were identified. These accessions are potentially useful sources for improving heat tolerance in cultivated wheat.	Hede *et al.* (1999)
Australia	Wheat variety ‘Lyallpur’	Despite favourable day/night temperature (18/13 °C), drought reduced kernel dry weight at anthesis.	Wardlaw (2002)
Sudan	Wheat variety ‘Debira’, ‘El Nelein’, ‘Donki’	A 2-year field study in two regions showed that ‘El Nelein’ performed best when sown late (air temp. 17–24 °C).	Ahmed *et al.* (2003)
China	Spring wheat variety ‘Ningchun18’	Soil water deficit both during the middle vegetative stage and the late reproductive stages and no-soil-water-deficit both during the late vegetative stage (booting) and the early reproductive stage (heading) had the highest yield increase of 25 and 14 %.	Zhang *et al.* (2006)
Egypt	Wheat cultivars ‘Sakha8’, ‘Sakha93’, ‘Sakha61’, ‘Chinese spring’	Based on drought susceptibility index ‘Sakha8'and ‘Sakha93’ were tolerant, and ‘Sakha61’ and ‘Chinese spring’ were susceptible.	El-Fadly *et al.* (2007)
Turkey	20 wheat cultivars (16 bread wheat, *Triticum aestivum*; four durum wheat, *Triticum durum* cultivars)	Considering drought sensitivity indices over 2 years, the bread wheat cultivars ‘Yayla-305’, ‘Gerek-79’, ‘Dagdas-94’ and ‘Bolal-2973’ were more drought-tolerant than other cultivars.	Bagci *et al.* (2007)
Argentina	Wheat, barley and Triticale	Wheat, barley and a Triticale cultivar were evaluated in three seasons under three thermal conditions: control and two heat conditions before anthesis. Stem elongation stage was most sensitive to high-temperature stress (yield reduction 46 %).	Ugarte *et al.* (2007)
Hungary	Wheat varieties ‘GK-Elet’, ‘Mv-Emese’	Pot culture experiment in growth chamber indicated that ‘Mv-Emese’ had better drought stress tolerance than ‘GK-Elet’.	Lukacs *et al.* (2008)
Bulgaria	Two drought-tolerant (‘Katya’, ‘Zlatitza’) and two drought-sensitive wheat varieties (‘Sadovo’, ‘Miziya’)	Drought-tolerant varieties ‘Katya’ and ‘Zlatitza’ had higher levels of these proteins, especially rubisco binding protein (RBP) and ATP-dependent calpain protease (Clp) proteins.	Demirevska *et al.* (2008)
USA	Spring wheat variety ‘Sinton’	Cool air temperature (18.33 °C) lengthened the lifespan and high temperature (26.11 °C) shortened the lifespan.	Frank *et al.* (2009)
Portugal	Four *Triticum* genotypes (‘Golia’, ‘Sever’, ‘Acalou’, ‘TE9306’)	Under heat stress (day/night 31/20 °C) during the grain-filling period, Fe and Mn helped to overcome stress.	Dias *et al.* (2009)
Iran	Wheat cultivars ‘Azar-2’, ‘Sardari’, ‘Frankia’, ‘Trakia’	‘Frankia’ performed better in various levels of terminal drought stress.	Dalirie *et al.* (2010)
Azerbaijan	Two bread wheat (‘Giymatli-2/17’, ‘Azamatli-95’) and two durum wheat variety (‘Garagylchyg-2’, ‘Barakatli-95’)	In drought-tolerant genotypes ‘Azamatli-95’ and ‘Barakatli-95’ this decrease was less pronounced than drought-sensitive ‘Garagylchyg-2’ and ‘Giymatli-2/17’.	Bayramov *et al.* (2010)
Pakistan	Four wheat genotypes viz., ‘LU-26s’, ‘Bhittai’, ‘Roshan’, ‘Taifu’	‘LU-26s’ performed best in drought, with minimum decrease in the growth parameters i.e. plant height, number of tillers and shoot biomass.	Shirazi *et al.* (2010)
Syria	Five genotypes	Late and very-late planting reduced yield traits significantly. Varieties were ranked based on tolerance to high-temperature stress: PBW 343 (susceptible) PBW 175 and HD 2865 (moderately susceptible) and HDR-77 and HD 2815 (tolerant).	Almeselmani *et al.* (2011)
Pakistan	Five wheat varieties (‘TJ-83’, ‘Imdad-2005’, ‘Abadgar-93’, ‘Moomal-2000’, ‘Mehran-89’)	‘Moomal-2000’ and ‘Mehran-89’ (better performed under 20–30 °C air temperature = heat stress). ‘TJ-83’, ‘Imdad-2005’ and ‘Abadgar-93’ were heat-sensitive.	Buriro *et al.* (2011)
Saudi Arabia	Three wheat genotypes (‘KSU-105’, ‘KSU-106’, ‘Yecora Roja’)	‘KSU-105’ performed better in late heat stress condition (25–30 °C). ‘KSU-106’ and ‘Yecora Roja' were heat-sensitive.	Refay (2011)
Russia	Eight wheat genotypes	‘Zlata’ showed sensitivity to low air temperature (−3 °C) and ‘Ester’ and Yubileinaya' showed tolerance to low temperature (−3 °C).	Karmanenko *et al.* (2011)
Ethiopia	18 wheat genotypes ‘B5-5B’, ‘S-17B’, and ‘WA-13’,‘Asassa, Bekelcha’, ‘Boohai, Egersa’, ‘Foka, Gerardo’, ‘Ilani, T Kilinto’, ‘Obsa, Oda’, ‘Quamy’, ‘Tob-66’, Yeror’, ‘CDSS93Y107’ ‘CD94523’	Water deficit significantly affected gas exchange and chlorophyll fluorescence parameters. It reduced the net photosynthesis rate, transpiration rate and stomatal conductance measured both at anthesis and grain-filling stages.	Bogale *et al.* (2011)
India	Wheat genotypes ‘HD2851, ‘HI8498’, ‘HDR77’, ‘PBW343’ and ‘HD2936’	Low temperature (<18–20 °C air temperature) during reproductive stage causes sterility of pollen grains. ‘HD2851, ‘HI8498'and ‘HDR77’ were highly affected by low temperature (<15 °C). Two other cultivars ‘PBW343’ and ‘HD2936’ were tolerant to low temperature (11.6–15 °C).	Chakrabarti *et al.* (2011)
United Kingdom	Wheat genotypes ‘Damani’, ‘Gomal-8’, ‘Hashim-8’, ‘DN-73’, ‘Zam-04’, ‘Dera-98’	‘Hashim-8’ was drought tolerant.	Khakwani *et al.* (2011)
Jordan	15 wheat genotypes: ‘Omguer-5’, ‘Genil-3’, ‘Stork’, ‘Korifla’, ‘Omrabi-5’, ‘Waha-1’, ‘Stojocri-3’, ‘Massara-1’, ‘Omsnima-1’, ‘Lagost-3’, ‘Heina’, ‘Ombar’, ‘Gersabil-2’, ‘Moulsabil-2’, and ‘Zeina-3’)	‘Waha-1’, ‘Omrabi-5’, and ‘Massara-1’ genotypes performed better in Mediterranean climate among genotypes studied.	Al-Karak (2012)
Russia	4 spring wheat (‘Zernagrad. 770’, ‘Sokol’, ‘Ratnik’, ‘Nutans’) and 2 barley (‘Saratov. 70’, ‘line 4’)	Among the 6 genotypes (wheat + barley) ‘Zernagrad. 770’ and ‘line 4’ were found to be stress tolerant (high temperature with drought).	Hossain *et al*. (2012*d*, *e*)

**Table 5:** Research findings related to the effect of temperature on wheat (*T. aestivum* L.) of Bangladesh studied by different scientists.

Types of research	Research findings	References
Effect of seeding time and spacing on yield of wheat under irrigated and non-irrigated conditions	Seeding of wheat should start first week of November and continue up to end of December.	Ahmed *et al.* (1976)
Effect of seeding dates on the yield and maturity of spring wheat	High temperature at the reproductive phase and low temperature at the early vegetative phase of wheat resulted in reduced number of days for attaining different phenological stages.	Hossain *et al.* (1987)
Influence of sowing dates and seed rate on two varieties of wheat	High temperature during tillering stage reduced tillers, resulted in low yield.	Hossain and Farid (1988)
Effect of dates of sowing and rate of fertilizers on yield of wheat under irrigated conditions	Late sown wheat affected by temperature twice: at germination stage and at reproductive stage.	Hossain *et al*. (1990)
Performance of some modern cultivars of wheat under different dates of planting	Delayed sowing reduced yield due to high-temperature stress at the grain-filling stage.	Sarker *et al.* (1996)
Drought stress effects on water relations of wheat	Drought stress significantly decreased the leaf water potential and relative water content of wheat, which had pronounced effects on photosynthetic rate.	Siddique *et al.* (2000)
Drought stress effect on photosynthetic rate and leaf gas exchange of wheat	In drought, photosynthetic rates decreased with a decrease in stomatal conductance. Tolerant varieties were less affected than sensitive ones.	Siddique *et al.* (1999)
Water-logging tolerance	Water logging is a widespread problem in irrigated and high rainfall wheat-growing regions of the world like Bangladesh.	Samad *et al.* (2001)
Kernel growth physiology of wheat under late planting heat stress	Reduced kernel size in heat-sensitive genotypes due to reduction in rapid kernel growth and duration.	Hasan and Ahmed (2005)
Spikelet sterility of wheat in farmer's field in northwest Bangladesh.	When sown 7 days earlier, wheat crops had more spikelet sterility than when sown 7 days later.	Saifuzzaman *et al.* (2008)
Effect of sowing dates on yield of wheat varieties and lines developed since 1998	Existing varieties may be sown November 15 to December 6 for good yield (3.5 tons ha^−1^).	Hossain *et al.* (2009)
Sowing dates mediated heat stress affects the leaf growth and dry matter partitioning in some spring wheat cultivars	It was clearly observed that high temperature caused a devastating effect on the leaf growth, dry matter partitioning and grain yield of wheat.	Ahamed *et al*. (2010)
Building yield in Bangladesh wheat crops: experience from traditional wheat-producing regions.	They identified that north region wheat get more time (105–110 days) to anthesis, due to long duration low temperature at winter (yield 4 tons ha^−1^). On the other hand, south region wheat needs 90–95 days to anthesis (yield 3 tons ha^−1^), due to high temperature in winter than north.	Rawson *et al.* (2011)
Resistance to rusts in Bangladeshi wheat	Considering on genetic basis, advance line ‘Francolin#1’ and the new variety ‘BARI Gom 26’ were found to be Ug99	Malaker and Reza (2011)
Farmers' preference and informal seed dissemination of first Ug99-tolerant wheat variety in Bangladesh	‘BAW1064’ (‘BARI Gom 26’) was more tolerant to rust Ug99 than other tested genotypes.	Pandit *et al.* (2011)
Evaluation of spring wheat under heat stress: I. Phenology, II. Growth and development	Due to high-temperature stress the stages of life cycle reduce their duration or length, which ultimately affected growth and development, finally yield.	Hossain *et al.* (2012*a*, *b*)
The effect of high-temperature stress on the phenology, growth and yield of five wheat genotypes	All the wheat genotypes where highly affected by high-temperature stress at late sown condition. Among these BARI Gom 26 were heat tolerant than others.	Hossain *et al*. (2012*c*)
Yield, protein and starch content of 20 wheat genotypes exposed to high temperature under late sowing conditions	Due to the high-temperature stress, yield and yield-related components, protein and starch contain of late-sown wheat were significantly affected.	Hakim *et al*. (2012)

**Table 6:** Research related to membrane thermostability, heat susceptibility, relative performance and stress stability study to identify heat-tolerant cultivars.

Wheat cultivars	Heat-tolerant cultivars	Susceptible cultivars	References
16 wheat varieties (‘Kalyansona/Mexi Pak’, ‘Sonora 64’, ‘INIA 66’, ‘Norteno 67’, ‘Sonalika’, ‘Nuri 70’, ‘Jupateco 73’, ‘Tonori 71’, ‘Balaka’, ‘Doel’, ‘Pavon 76’, ‘Ananda (BAW 18)’, ‘Kanchan (BAW 28)’, ‘Akbar (BAW 43)’, ‘Barkat (BAW 39)’, ‘Aghrani (BAW 38)’ and two advance lines (‘BAW 171’, BAW 452’)	‘Kanchan’ and two advanced lines ‘BAW 171’ and ‘BAW 452’ were high-yielding and matured early.	Other cultivars showed sensitivity to high temperature when sown late.	Razzaque *et al.* (1994)
Sonilika’, ‘Balaka’, ‘Ananda’, ‘Kanchan’, ‘Akbar’, ‘Barkat’	‘Kanchan’ performed best in all sowing conditions.	Other cultivars showed sensitivity to high temperature (28–32 °C).	Mannaf (1995)
‘Ananda’, ‘Pavon’, ‘Aghrani’, ‘Barkat’, ‘Akbar’, ‘Kanchan’, ‘Protiva’, ‘Balaka’, ‘Sawgat’, ‘Sonora’	Based on a membrane thermo-stability test, ‘Ananda’, ‘Pavon’, ‘Aghrani’ and ‘Barkat’ were classified as relatively heat tolerant while ‘Akbar’, ‘Kanchan’ and ‘Protiva’ were moderately tolerant.	The remaining three varieties ‘Balaka’, ‘Sawgat’ and ‘Sonora’ had the shortest heat killing time and were considered to be heat sensitive.	Hossain *et al.* (1999), Sikder *et al.* (2001)
Two wheat cultivars: ‘Kanchon’, ‘Sonora’	Based on membrane thermo-stability and contribution of pre-anthesis reserves.	‘Kanchan’ was considered to be relatively heat tolerant and ‘Sonora’ as heat sensitive.	Uddin *et al.* (2005)
‘Agrani’, ‘Kanchan’, ‘CB-30’, ‘Sonora’	Based on praline content, membrane thermo-stability and heat susceptibility index, ‘Agrani’, ‘Kanchan’ and ‘CB-30’ were heat tolerant.	‘Sonora’ was heat sensitive.	Hasan *et al.* (2007)
10 wheat genotypes (‘Kanchan’, ‘BAW-969’, ‘SW89-5124*21FASAN’, ‘W 82/VEE/KOEL 1 3/PEG//MLR/BUC’, ‘HP/ 1731, SERI/ RAYON’, ‘LAJ 3302/2* M088’, ‘BORL 95/ LAJ 3302’, ‘OASS/SKAUZ// 4*BCN’ and ‘SCAN’)	Based on relative performance and heat susceptibility index MW-8, BW-4 and BW-3.	Other 7 cvs. were heat susceptible.	Rahman *et al.* (2009)
‘Agrani’, ‘Prodip’, ‘Bijoy’, ‘CB 69’, ‘Sourav’, ‘Sufi’, ‘BAW 1064’, ‘Gourab’, ‘Kanchan’, ‘Shatabdi’, ‘CB 30’, ‘Sonora’, ‘CB 24’, ‘CB34’, ‘Protiva’	Based on cell membrane thermo-stability, photosynthates stem reserve translocation, ‘Agra, ‘Kanchan’, ‘CB 30’ and ‘CB 69’ were found as heat-tolerant genotypes for cultivation in Patuakhali district.	‘Sonora’, ‘CB 34’, ‘CB 24’ and ‘Protiva’ were heat-sensitive genotypes and remaining seven were heat susceptible.	Haque *et al*. (2009)
‘Gourab’, ‘Sourav’, ‘Kanchan’, ‘Shatabdi’, ‘Sonora’, ‘Kalyansona’	Based on the growing degree days, helio-thermal unit, heat use efficiency and pheno-thermal index, ‘Gourab’, ‘Sourav’, ‘Kanchan’ and ‘Shatabdi’ performed better in heat stress.	Heat-sensitive cultivars, Sonora and ‘Kalyansona’ showed sensitivity to heat stress.	Sikder (2012)
10 spring wheat genotypes ‘Gen/3/Gov’, ‘PB81/PVN’, ‘Fang 60’, ‘Kanchan’, ‘Sari 82’, ‘HI 977’, ‘HAR 424’ ‘PF 70354’, ‘Opata’, ‘Fyn/Pvn’	Considering canopy temperature depression, ‘Gen/3/Gov’ seemed to be the best entry for late planting with reasonably high yield, moderate grain size and growth period.	Other varieties did not show better performance in late heat stress.	M. A. Rahman *et al.* (2009); M. M. Rahman *et al.* (2009)
‘Sourav’, ‘Gourab’, ‘Shatabdi’, ‘Sufi’, ‘Bijoy’, ‘Prodip’, ‘BAW 1059’, ‘BAW 1064’	According to stability analysis ‘Sourav’ was a stable variety in all environmental conditions.	‘Prodip’ and ‘BAW1064’ were sensitive genotypes.	Kabir *et al.* (2009)
‘Gourab’, ‘Sourav’, ‘Kanchan’, ‘Shatabdi’, ‘Sonora’, ‘Kalyansona’	Based on membrane thermo-stability in a laboratory test, then canopy temperature depression and stem reserve mobilization capacity, ‘Shatabdi’, ‘Sourav’, ‘Kanchan’ and ‘Gourab’ were grouped as heat tolerant.	Whereas cultivars ‘Sonora’ and ‘Kalyansona’ were considered as heat sensitive.	Sikder and Paul (2010)
20 wheat genotypes	Based on the % cell membrane injury and seedling proline content ‘Bijoy’, ‘Sufi’, ‘Kanchan’, ‘Fang 60’, ‘BAW 1059’, ‘BL 1883’, ‘BL 1022’, ‘IVT 7’, ‘IVT 8’, ‘IVT 9’, ‘IVT 10’ and ‘BAW 917’ were heat-tolerant ().	Remaining 8 genotypes were heat sensitive.	Ahmed and Hasan (2011)
‘Sourav’, ‘Gourab’, ‘Shatabdi’, ‘Sufi’, ‘Bijoy’, ‘Prodip’, ‘BARI Gom 25’, ‘BARI Gom 26’	Both in too early and very late heat stress conditions, genotypes ‘Sourav’ and ‘BARI Gom 25’ were heat tolerant.	Variety ‘Prodip’ showed sensitivity to high temperature.	Hossain *et al.* (2011)
‘Gourab’, ‘BARI Gom 25’ and ‘BARI Gom 26’	Considering on heat susceptibility and relative performance, variety ‘BARI Gom 25’ was the best-performing variety, followed by ‘BARI Gom 26’	‘Gourab’ was susceptible variety.	Hossain and Teixeira da Silva (2012)

Problems of salinity and water logging, heavy erosion of soils and riverbanks all accelerate soil degradation. Estimates of areas affected by nutrient depletion and other forms of soil degradation are ∼5.6 million ha in Bangladesh with ∼0.83 million ha of land being affected by various degrees of salinity. With climate change, saline water may extend to currently non-saline land, ultimately affecting (negatively) crop production (Rahman 2011). The Bangladeshi Ministry of Agriculture has also initiated a number of agricultural programmes, such as the development and distribution of drought- and saline-resistant crop varieties to enhance year-round production. Several projects are focused on improving the infrastructural facilities for varietal development to combat these impacts of climate change. For example, the Wheat Research Centre in Bangladesh has so far released 28 wheat varieties. Of these, BARI Gom 23 (‘Bijoy’) and BARI Gom 24 (‘Prodip’) released in 2005, BARI Gom 25 and BARI Gom 26 released in 2010, and BARI Gom 27 and BARI Gom 28 released in 2012 are recommended for commercial cultivation (Fig. 5). All these varieties are high yielding and heat tolerant, resistant to leaf rust and tolerant of bipolaris leaf blight. Of these varieties, BARI Gom 25 tolerates a moderate level of salinity while BARI Gom 26 and BARI Gom 27 are moderately resistant to the Ug99 race of stem rust. Moreover, BARI Gom 28 matures very early and is highly tolerant of terminal heat stress (BARI 2012*b*). The major stress faced by wheat in South Asia is high temperature, mainly terminal heat stress (Joshi *et al*. 2007*a*), which was defined by Fischer and Byerlee (1991), due to mean daily temperature above 17.5 °C in the coolest month. Both the proximity to the equator and popular cropping systems, which involve the late sowing of wheat, are the major causes of exposure of wheat in Bangladesh and other neighbouring countries to high temperatures during grain filling (Rane *et al*. 2000). Therefore, breeding for high-temperature tolerance in wheat is another major objective of wheat improvement programmes in South Asian countries, including Bangladesh, India and Nepal. Besides the development of varieties and production initiatives, CIMMYT and WRC will strengthen their breeder seed production capacity in the next 5 years to meet the increased demand of climate-vulnerable areas.

### Impact of climate on wheat diseases and their related research

The two major characteristics of the eastern part of South Asia are high temperatures and humidity (Joshi *et al*. 2007*c*). This leads to two important stresses: heat, and spot blotch disease caused by *Bipolaris sorokiniana* (Sacc.) shoem syn. *Drechslera sorokiniana* (Sacc.) Subrm and Jain (syn. *Helminthosporium sativum*, teleomorph *Cochliobolus sativus*) (Joshi *et al*. 2004*a*, *b*; Pandey *et al*. 2005; Malaker and Reza 2011). *Septoria* leaf blotch can also severely damage yield (Mbarek and Teixeira da Silva 2011). Saari (1998) reported that the average yield losses due to leaf blight in the Indian subcontinent were as much as 17.5 %. It is generally believed that the level of resistance in high-yielding wheat genotypes is still unsatisfactory and needs to be improved significantly in warmer humid regions of South Asia (Joshi *et al*. 2007*a*, *b*, *c*). Consequently, an integrated approach, with host resistance as a major component, is generally considered best for controlling the disease (Joshi and Chand 2002). In this context, CIMMYT is working with different research institutes in South Asia to develop cultivars that are suitable for these regions.

In Bangladesh, mean temperature in winter (rabi) has risen by 0.66 °C since 1990 and a further warming of 2.13 °C by 2050 is predicted (Poulton and Rawson 2011). A rise in temperature can be expected to increase the severity of *Bipolaris* leaf blight and other wheat diseases in the future because a warm and humid climate favours the development and spread of the pathogen. Changes in the virulence pattern of this pathogen rendering increased susceptibility of wheat varieties are also not unlikely. Increasing temperature might also favour the development and adaptation of wheat stem rust, which can tolerate higher temperatures than other rusts. Although it has not been critically investigated or ascertained, a global rise in temperature could promote mutation and the development of new virulent races of rust pathogens.

Diseases, particularly rusts, are one of the major constraints to sustainable production of wheat worldwide (Saari and Prescott 1985), but in Bangladesh, leaf rust is the second most important disease (caused by *Puccinia triticina* Eriks.) of wheat after *Bipolaris* leaf blight [caused by *B. sorokiniana* (Sacc. in Sorok.) Shoem] (Malaker and Reza 2011). In this context, a total of 183 wheat genotypes consisting of crossing block materials, advanced breeding lines and varieties released in Bangladesh were tested for seedling resistance to rusts at CIMMYT in 1994 and at DWR (Directorate of Wheat Research) Shimla, India, in 1996 and 2003 (Malaker and Reza 2011). Available advanced lines and breeding materials have also been evaluated at Kenya Agricultural Research Institute in Kenya since 2006 for their reaction against the Ug99 (TTKSK) race of stem rust under epiphytotic conditions. It has been observed that most of the existing Bangladesh varieties are resistant to *Bipolaris* leaf blight. On the other hand, under Bangladesh's agro-climatic conditions, the first incidence of leaf rust is usually observed in mid-February and its severity increases between mid- and late March (Malaker and Reza 2011). Wheat planted at the optimum time either escapes the disease to a large extent, or suffers less than a wheat crop planted late in the crop season. Yield losses due to leaf rust are usually <10 %, but can be 30 % or more depending on the level of susceptibility, environmental conditions and the stage of crop development at the initial stage of infection (Singh *et al*. 2002). Although the losses due to leaf rust in the currently cultivated wheat varieties have not been calculated, they may be significant under favourable conditions for disease development, particularly if infection occurs early in the crop season or if a susceptible variety is sown late (Malaker and Reza 2011). Wheat stem rust caused by *Puccinia graminis* Pers. f.sp. *tritici* Eriks. & E. Henn. occurred in Bangladesh during the early years of wheat development. However, the disease has not been observed in Bangladesh since the mid-1980s, possibly due to the introduction of several resistant varieties (Malaker and Reza 2011). Yellow rust of wheat caused by *Puccinia striiformis* West. f.sp. *tritici* Eriks. & E. Henn. occurs occasionally with lowtomoderate severity and is restricted to the north-western districts only, where relatively cooler climates prevail during the winter months. However, both stem and yellow rusts remain a major problem in some of the wheat zones around the world (Malaker and Reza 2011). Most of the released Bangladesh wheat varieties are resistant to rust (leaf, stem and yellow rust). A total of seven leaf rust resistance genes (*Lr1*, *Lr3*, *Lr10*, *Lr13*, *Lr23*, *Lr26* and *Lr34*), seven stem rust resistance genes (*Sr2*, *Sr5*, *Sr7b*, *Sr8b*, *Sr9b*, *Sr11* and *Sr31*) and two yellow rust resistance patterns (Yr2KS and Yr9) were postulated to be present in existing varieties. The gene *Lr13* was found to be the most frequent leaf rust resistance gene in var. BARI Gom 25 (released in 2010), and present in combination with other *Lr* genes in seven other varieties (Table 7). Genes *Lr26* and *Lr1* were detected in four and five varieties, respectively. Genes *Lr10*, *Lr23 and Lr3* were postulated as present in three, two and one varieties, respectively. Out of six *Lr* genes, combinations of only two genes were inferred in most of the varieties (Table 7). Clear leaf tip necrosis indicated the presence of the adult plant slow leaf rusting resistance gene *Lr34* in vars. Sourav and BARI Gom 26 (Malaker and Reza 2011).

**Table 7:** Postulated rust resistance genes in the released wheat varieties of Bangladesh (source: Malaker and Reza 2011).

Variety	Year of release	Postulated rust resistance genes		
		*Lr*	*Sr*	*Yr*
Kanchan	1983	*Lr13*+ *Lr23*	R^a^	–
Akbar	1983	*Lr10*+ *Lr13*	–	–
Ananda	1983	*Lr1*+ *Lr13*	–	–
Barkat	1983	*–*^b^	*Sr2*+ *Sr8b*+ *Sr9b*+ *Sr11*+	–
Aghrani	1987	*Lr10*+ *Lr13*	–	–
Protiva	1993	*Lr13*+ *Lr23*	–	–
Sourav	1998	*Lr1*+ *Lr26*+ *Ltn*+^c^	*Sr5*+ *Sr31*+	*Yr9+*
Gourab	1998	*Lr1*+ *Lr26+*	*Sr5*+ *Sr31*+	*Yr9+*
Shatabdi	2000	*Lr1*+ *Lr13+*	*Sr8b*+ *Sr9b*+ *Sr11*+	*Yr2KS*
Sufi	2005	*Lr3*+ *Lr26*	*Sr2*+ *Sr31*+	*Yr9+*
Bijoy	2005	*–*	R	*–*
Prodip	2005	*Lr1*+ *Lr26*+	*Sr2*+ *Sr5*+ *Sr31*+	*Yr9+*
BARI Gom 25	2010	*Lr13+*	R	*–*
BARI Gom 26	2010	*Lr10*+ *Lr13*+ *Ltn*+	*Sr8b+ Sr9b+ Sr11+* Moderate level of APR to Ug99 race of stem rust	–
BARI Gom 27^d^	2012	–	*Sr2+?* Good level of APR to Ug99 race of stem rust	–
BARI Gom 28^d^	2012	–	–	–

R, resistant; APR, adult plant resistance.

^a^Resistant to all Indian pathotypes of stem rust.

^b^Genes were not postulated.

^c^Leaf tip necrosis gene *Lr34*.

^d^Rust resistance genes present in these two varieties have not yet been identified (may have been detected in the source countries, but we do not have the information); presence of *Sr2* (adult plant slow rusting stem rust resistance gene) in BARI Gom 27 is to be confirmed.

The danger posed by the Ug99 strain of stem rust to global wheat production is widely recognized, including in Bangladesh. The release, in 2010, of the first Ug99-resistant wheat variety (BARI Gom 26—previously known as BAW, and popularly called Hashi) was a major step to countering the threat of this disease in Bangladesh. Subsequently, the variety Francolin (also known as BARI Gom 27 or BAW 1120) was released in March 2012. Originating from CIMMYT-Mexico, Francolin possesses good resistance to all variants of Ug99 along with impressive agronomic performance, yielding ∼10 % more than the most popular variety Shatabdi in 3 years of multi-location testing in Bangladesh (CIMMYT 2012).

## Conclusions and forward look

About 80 % of people in Bangladesh depend directly on agriculture for their food and livelihood, with wheat being the second most important crop after rice. However, climate change leading to global warming has already produced a radical change in temperature regimes in Bangladesh. This will impact strongly on wheat production. The present review provides the first up-to-date perspective and detailed analysis of wheat research in Bangladesh, and the impact that global warming will have on its agriculture, especially wheat farming. This will allow wheat breeders to plan to mitigate the effects of further global warming. This will have direct implications for food security in Bangladesh as well as South Asia. The development of stress-tolerant wheat within the wider context of a breeding programme, particularly with respect to drought and flooding, needs to be supported by *in vitro* protocols (Farshadfar *et al*. 2012) as well as molecular techniques (Chaabane *et al*. 2012; Diab *et al*. 2012), while genetic modification of crops to increase photosynthesis or metabolomics needs greater focus in wheat improvement programmes (Kershanskaya and Teixeira da Silva 2010; Ruan and Teixeira da Silva 2011; Ruan *et al*. 2012).

## Contributions by the authors

Both authors have contributed equally to this manuscript.

## Conflict of interest statement

None declared.
